# Increased IgG but normal IgA anti-pneumococcal protein antibodies in lung of HIV-infected adults^☆^

**DOI:** 10.1016/j.vaccine.2013.04.062

**Published:** 2013-08-02

**Authors:** Andrea M. Collins, Sherouk El Batrawy, Stephen B. Gordon, Daniela M. Ferreira

**Affiliations:** a Liverpool School of Tropical Medicine, Liverpool L3 5QA, UK; b The Royal Liverpool and Broadgreen University Hospital, Liverpool L7 8XP, UK

**Keywords:** *Streptococcus pneumoniae*, PspA, Pneumolysin (Ply), Bronchoalveolar lavage (BAL), HIV infection, Pneumococcal colonization

## Abstract

PspA and pneumolysin (Ply) are important protein vaccine candidates. HIV infection is associated with increased susceptibility to pneumococcal pneumonia and concomitantly high pneumococcal carriage rates. Pneumococcal exposure is immunizing at the mucosa in healthy adults and so we wished to determine if the increased pneumococcal exposure in HIV-infected adults would be associated with altered pneumococcal specific antibody responses. We measured serum and bronchoalveolar lavage (BAL) fluid immunoglobulin (Ig)G and IgA to PspA and Ply in HIV-infected and healthy age-matched adults.

Naturally generated anti-Ply and anti-PspA IgG levels but not IgA were significantly increased in HIV-infected subjects in BAL independent of the hyperglobulinaemia commonly associated with HIV. There was therefore no evidence of a defect in mucosal responses to pneumococcal protein antigens among HIV-infected adults.

With regard to future vaccination strategies, simply increasing mucosal anti-pneumococcal protein Ig levels, without addressing functional protective response, is not likely to be effective in preventing pneumococcal pneumonia in HIV-infected individuals.

## 1 Introduction

*Streptococcus pneumoniae* is the most common cause of community acquired pneumonia in both HIV-infected and non-HIV infected individuals [1]. The rate of invasive pneumococcal disease (IPD) in HIV-infected individuals is 40–100 times greater than age-matched controls, due to several immune defects including low CD4 T-cell numbers and impairment of the humoral response [2]. The factors underlying the susceptibility of HIV-infected patients to pneumococcal pneumonia remain poorly understood.

Despite the fact that the 7-valent pneumococcal conjugate vaccine (PCV) has shown up to 75% protection against recurrent IPD from vaccine serotypes in HIV-infected individuals [3], alternative protective strategies including protein vaccines remain an important worldwide priority, due to serotype-replacement, a lack of longevity of effect and an unclear protection against pneumonia.

Pneumococcal surface protein A (PspA) and pneumolysin (Ply) are leading protein vaccine candidates able to confer protection against pneumonia and invasive disease in murine models [4]. Antibodies to PspA and Ply appear early in life and the increase of antigen-specific IgA and IgG levels with age suggests a role for these responses in the prevention of IPD [5]. Moreover, colonization in children and healthy adults elicited antibody responses to both antigens [6,7].

HIV-infected African adults have high rates of pneumococcal colonization and a persistently poor control of pneumococcal colonization [8]. Frequent carriage is likely to be associated with repeated antigenic stimulation and therefore high antibody levels. We tested the hypothesis that pneumococcal protein-specific (PspA and Ply) responses would be altered in HIV-infected patients and would be either elevated due to increased rates of pneumococcal carriage and disease, or reduced due to their immunocompromised state. We also postulated that the response would be compartmentalized and mucosal and serum antibody levels would be independently regulated.

We have previously reported higher levels of serum anti-Ply in HIV-infected patients compared to HIV-non infected controls [9]. High levels of anti-pneumolysin IgG did not predict protection from invasive pneumococcal disease or indicate that an effective immune response has occurred in HIV infected patients. Others have reported that impaired immunity to Ply was associated with a higher incidence of pneumococcal bacteraemia in HIV-infected patients [10]. Levels of specific anti-pneumococcal protein IgG and IgA in lung fluid have not been reported previously.

We have now measured anti-PspA and anti-Ply IgG and IgA in the lung and serum of HIV-infected patients and an HIV-non infected control group.

## 2 Subjects and methods

### 2.1 Patient selection and HIV testing

50 volunteers were recruited after obtaining fully informed written consent at the Queen Elizabeth Central Hospital, Blantyre, Malawi. No volunteer had any history of previous pneumococcal disease or vaccination. The study was given ethical approval by the Liverpool School of Tropical Medicine and the College of Medicine (University of Malawi) Research Ethics Committees. HIV status was determined by HIV gold and HIV serocard: Trinity Biotech and ELISA. All subjects were asymptomatic (normal physical examination and chest radiograph).

### 2.2 Bronchoscopy and bronchoalveolar lavage (BAL)

Bronchoscopy and BAL were performed as previously described [11] using 200 ml of warm saline. After centrifugation at 330 g for 10 min BAL supernatant was transferred into 50 ml centrifuge tubes and stored at–80 °C before ELISA testing.

### 2.3 ELISA measurement of Ig levels

ELISA assays were performed to determine total IgG and pneumococcal specific IgG and IgA as previously described by our group [11,12]. Microplates (NUNC) were coated with Goat anti-human IgG (Dako) for total IgG or 2 μg/ml of PspA or Ply for pneumococcal specific IgG. Serial diluted samples and a standard sample with known concentration of total IgG were added in duplicate. Antibody detection was performed by goat anti-human IgG or IgA biotinylated antibody followed by streptavidin alkaline phosphatase antibody (Oxford Biotechnology). 0.5 mg/ml of *p*-nitrophenyl phosphate (PNPP) (Sigma) was used for development. Absorbance was read at 405 nm using a FLUOstar Omega (BMG Labtech). Results were analyzed using 4-parameter fitted curve and samples that showed CV > 15% were repeated.

### 2.4 Statistical analysis

Mean and 95% confidence intervals of Ig levels are presented at the graphs and log transformed data was used for statistical analysis. Ratios of pneumococcal specific IgG to total IgG were calculated on log transformed data. Unpaired Student *t*-test was used to compare levels between HIV-infected and control groups. Pearson's *r* test was used to analyze correlation between BAL and serum concentrations. *p* ≤ 0.05 was considered statistical significant.

## 3 Results

25 HIV-infected and 25 HIV negative (age and sex matched) volunteers were recruited. No volunteers were receiving highly active anti-retroviral therapy (HAART). The median CD4 count was 340 cells/mm^3^ in the HIV-infected and 785 cells/mm^3^ in the HIV negative subjects. The BAL volume yield ranged from 72 to 148 ml. No serious adverse events related to the research bronchoscopy were observed.

**Fig. 1:**
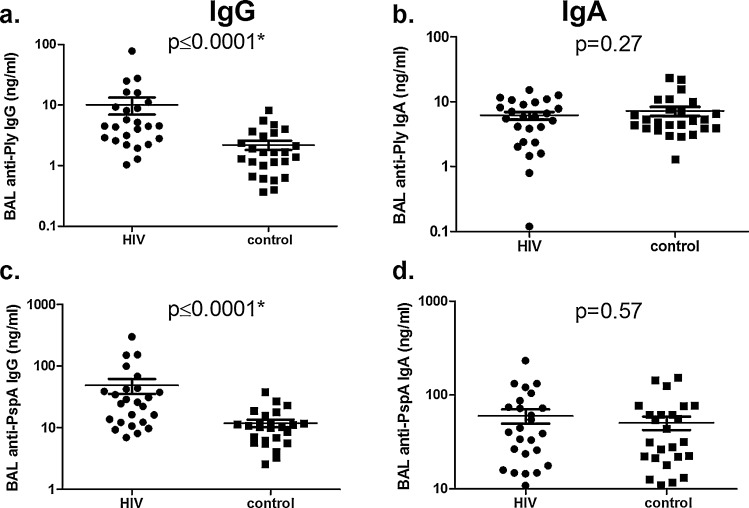
Pneumococcal specific Ig concentrations in bronchoalveolar lavage (BAL) of HIV-infected and control groups. Each plot represents a subject and bars are the mean of values and 95% confidence intervals. Anti-Ply IgG (a), Anti-Ply IgA (b), Anti-PspA IgG (c) and Anti-PspA IgA (d), levels of HIV-infected and control groups are expressed in ng/ml. *Statistical significance using unpaired Student's *t*-test (*p* < 0.05).

We observed that anti-Ply and anti-PspA IgG levels in BAL were significantly higher in the HIV-infected subjects compared to the control subjects (*p* < 0.0001) (Fig. 1a and c). No difference was observed for anti-Ply and anti-PspA IgA levels in BAL (Fig. 1b and d). We measured total IgG in BAL and also expressed anti-Ply and anti-PspA IgG levels as ratios of total IgG (Table 1). The HIV-infected group had higher total IgG levels than the control group (mean, 95%CI; 13,404 ng/ml, 6985–19823 vs 4400 ng/ml, 3254–5546, *p* = 0.003 using un-paired *t*-test). Higher ratios of pneumococcal specific IgG to both antigens were observed in BAL of HIV-infected subjects than control group (For Ply: 0.18, 0.13–0.23 vs 0.05, 0.007–0.09, *p* = 0.0003 and for PspA: 0.36, 0.31–0.41 vs 0.27, 0.23–0.30, *p* = 0.001).

**Fig. 2:**
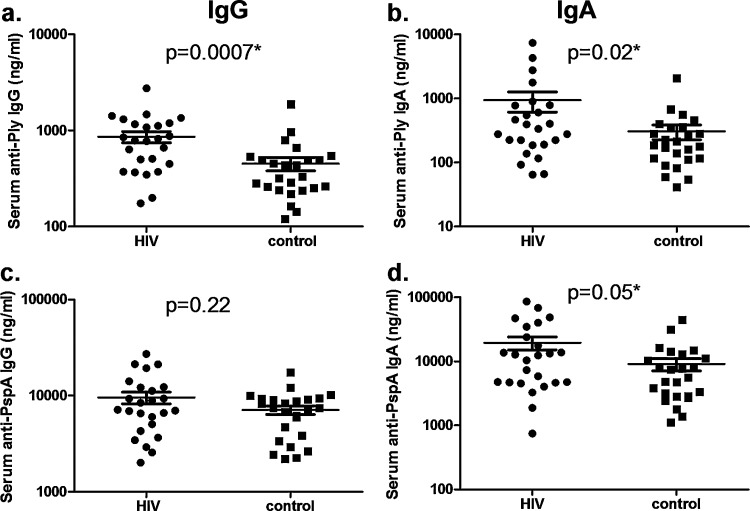
Pneumococcal specific Ig concentrations in serum of HIV-infected and control groups. Each plot represents a subject and bars are the mean of values and 95% confidence intervals. Anti-Ply IgG (a), Anti-Ply IgA (b), Anti-PspA IgG (c) and Anti-PspA IgA (d), levels of HIV-infected and control groups are expressed in ng/ml. *Statistical significance using unpaired Student's *t*-test (*p* < 0.05).

The HIV-infected group also showed increased serum anti-Ply IgG (*p* = 0.0007) and IgA (*p* = 0.02) (Fig. 2a and b) slightly increased anti-PspA IgA (*p* = 0.05, Fig. 2d) but no increase in anti-PspA IgG (*p* = 0.22, Fig. 2c).

Anti-Ply and Anti-PspA IgG and IgA levels were much lower in BAL than serum in all subjects; even considering the dilution of BAL samples (diluted 60–100-fold during collection).

We observed a generally poor correlation between BAL and serum levels of anti-Ply IgG (HIV: *r* = −0.01, *p* = 0.92 and control: *r* = 0.02, *p* = 0.89); anti-Ply IgA (HIV: *r* = 0.11, *p* = 0.59 and control: *r* = −0.04, *p* = 0.81); anti-PspA IgG (HIV: *r* = −0.04, *p* = 0.83 and control: *r* = 024, *p* = 0.24) and anti-PspA IgA (control: *r* = 0.11, *p* = 0.59). A statistical significant correlation between BAL and serum was observed to anti-PspA IgA in HIV-infected subjects only (*r* = 0.41, *p* = 0.03).

## 4 Discussion

We have shown that HIV-infected patients had higher levels of pneumococcal specific IgG (anti-Ply and anti-PspA) in BAL than levels measured in a control group. This effect may be attributed to polyclonal B-cell activation and hyperglobulinaemia but it is not uniform as we have demonstrated increased ratios of pneumococcal specific IgG in relation to total IgG in BAL.

Serum immunoglobulins (Ig) are critical in defence against invasive disease and lung-lining fluid Ig are critical in primary mucosal defence, particularly to opsonophagocytosis of capsulate pneumococci by alveolar macrophages [13].

In HIV infection there is poor correlation between total and specific Ig levels in serum to encapsulated bacteria because of the associated polyclonal B-cell activation [14]. The pulmonary mucosal surface, however, does not exhibit the same immune defects as does the systemic defence. Pulmonary mucosal Ig responses to protein antigen are locally regulated and independent of serum IgG levels [15] with some mucosal immunity relatively preserved, compared with systemic immunity [11,16].

In this study, a poor correlation was found between serum and BAL pneumococcal specific Ig levels in both HIV-infected and control groups. This suggests that local synthesis rather than transudation of antibody from serum into the alveoli is more likely to be the cause of the increased pneumococcal-specific IgG in BAL. Pneumococcal colonization of the upper airway may lead to micro aspiration of bacteria to the lungs leading to antigen uptake by antigen-presenting cells and local production by mature memory B cells in the lung [17].

The fact that we observed increased IgG but not IgA in the lung of HIV-infected subjects is supported by our previous published work showing that intra-nasal exposure to pneumococcus increases pneumococcal specific IgG but not IgA in the lung of healthy adults [12]. The absence of difference in IgA levels in this study suggests that HIV infection does not directly alter IgA levels and that increased carriage of pneumococci in HIV infected subjects does also not increase BAL IgA. We conclude that there is therefore no lung defect in anti-protein IgA responses but rather there are different roles in protection mediated by IgG and IgA in the lung compartment.

HIV-infected African adults have increased risk of repeat colonization [18], persistently poor control of pneumococcal colonization and as a result high rates of colonization compared to non-infected adults [8]. We propose that increased IgG responses observed in BAL in the HIV-infected group are a consequence of their higher rates of carriage and previous infection. The functional significance of this natural boosting effect as well as the mechanism underlying the defective mucosal protection from carriage is still to be elucidated. We have previously reported high anti-pneumococcal polysaccharide specific (PPS) Ig but deficient opsonophagocytic activity (OPA) function [19,20] in BAL of HIV-infected patients. Other explanations include mucosal T cell responses or loss of other local regulatory constraints due to the HIV infection.

To further understand the function of BAL anti-pneumococcal protein responses, future studies will require microbiological data related to episodes of documented carriage and disease. Functional assays should also be investigated although specific pneumococcal protein antigen OPA assays are not currently available. The humoral response is however not the only factor involved in protection from mucosal disease but part of an interplay of various immune mechanisms affected by HIV, including the critical role of CD4 TH17 cells.

In conclusion, this study reveals that specific anti-pneumococcal protein IgG and IgA levels are differentially regulated according to compartment and are maintained during HIV-infection. The implications of this study are that vaccination resulting only in increased levels of anti-protein IgG is unlikely to be effective at preventing pneumococcal disease in HIV-infected adults.

## Figures and Tables

**Table 1:** BAL levels of total IgG, anti-pneumococcal specific IgG and ratios of pneumococcal specific IgG: total IgG in HIV-infected and control groups.

	HIV-infected	Control	*p*-Value
Total IgG	13,404 (6985, 19,823)	4400 (3254, 5546)	0.003^a^
Anti-Ply IgG	9.83 (2.70, 16.97)	2.19 (1.40, 2.97)	0.02^a^
Anti-Ply:total IgG ratio	0.18 (0.13, 0.23)	0.05 (0.01, 0.09)	0.0002^a^
Anti-PspA IgG	45.66 (17.99, 73.34)	11.82 (8.49, 15.14)	0.01^a^
Anti-PspA:total IgG ratio	0.36 (0.31, 0.41)	0.27 (0.24, 0.30)	0.001^a^

Values are mean ± 95% confidence intervals and ratios of pneumococcal specific IgG:total IgG.

a Using unpaired students’ *t*-test, *p* ≤ 0.05 when comparing HIV-infected and control groups.
